# Steroid Refractory Autoimmune Haemolytic Anaemia Secondary to Sarcoidosis Successfully Treated with Rituximab and Mycophenolate Mofetil

**DOI:** 10.1155/2016/9495761

**Published:** 2016-08-02

**Authors:** Sarah Green, Erica Partridge, Edore Idedevbo, Anton Borg

**Affiliations:** Warwick Hospital, Lakin Road, Warwick, Warwickshire CV34 5BW, UK

## Abstract

Autoimmune haemolytic anaemia is not a well-recognised complication of sarcoidosis. We describe the case of a 30-year-old female who presented with acute warm haemolytic anaemia and widespread lymphadenopathy. Sarcoidosis was diagnosed on lymph node biopsy and further investigation. The haemolytic anaemia responded only to a high dose of steroids. Evidence regarding treatment of steroid refractory autoimmune haemolysis secondary to sarcoidosis is lacking. Based on the emergent evidence that both disorders share common immunopathogenic mechanisms involving Th1 and Th17 lymphocytes, our patient was given rituximab and mycophenolate mofetil to successfully suppress the haemolysis and sarcoid activity.

## 1. Introduction 

Sarcoidosis is a multisystem granulomatous disease of unknown aetiology. The evidence is suggestive of an exaggerated granulomatous reaction which occurs in genetically susceptible individuals following exposure to a yet unidentified antigen [1]. It commonly presents with lung involvement such as pulmonary fibrosis, skin lesions such as erythema nodosum, eye involvement, and lymphadenopathy. It rarely affects the heart, kidneys, nervous system, and parotid gland. Spontaneous resolution occurs over a period of 2 years in 50% of patients. Prognosis is dependent on the severity of critical organ involvement. Mortality is higher than in the general population due to sarcoidosis itself [2].

Presentation with autoimmune haemolytic anaemia is not well recognised in sarcoidosis. Autoimmune haemolytic anaemia occurs when autoantibodies target antigens on red blood cells. It affects 1 to 3 in 100,000 people per year and it can be life-threatening [3]. It may be secondary to malignant lymphoproliferative disorders, viral infections, drugs, and other autoimmune disorders. Most cases are idiopathic. The presentation of haemolysis is typically with anaemia and/or jaundice.

## 2. Case Presentation

A 30-year-old previously healthy female presented with a 2-day history of progressive dizziness, shortness of breath, jaundice, and abdominal pain. Over the last 6 months, the patient had lost 3 stones in weight which she attributed to dieting. She was not taking medications. There was no family history of anaemia, jaundice, or gallstones.

Clinically, the patient was jaundiced and had left submandibular lymphadenopathy and splenomegaly.

Initial blood tests revealed severe spherocytic haemolytic anaemia (see Table 1). A red cell enzyme deficiency screen, including G6PD, was done once the haemolysis was subdued with treatment. Flow cytometry screening for paroxysmal nocturnal haemoglobinuria and basic Coombs tests were negative. A super Coombs done later was positive for warm IgG autoantibodies. A viral screen including HIV, EBV, and CMV and a mycoplasma serology were negative. Immunoglobulin levels were increased but the paraprotein was absent. Autoimmune and vasculitic screens, including antinuclear, anti-extractable nuclear, and anti-neutrophil cytoplasmic autoantibodies, were absent. A CT of the neck, abdomen, and pelvis showed extensive neck, mediastinal, and upper abdominal lymphadenopathy and significant splenomegaly. As lymphoma was suspected, a cervical lymph node biopsy was taken, which showed noncaseating and nonnecrotising granulomatous lymphadenitis. The ratio of CD4 : CD8 T-lymphocytes in lymph node biopsy was 5.8 on flow cytometry analysis and 4.0 on immunohistochemistry. Sarcoidosis was diagnosed on exclusion of other causes of such lymphadenitis. The bone marrow was normal other than for erythroid hyperplasia. There was no evidence of pulmonary involvement on the CT scan and pulmonary function tests. Serum angiotensin-converting enzyme (ACE) was elevated and adjusted serum calcium was borderline high. Alkaline phosphatase and 1,25-dihydroxyvitamin D levels were normal.

The patient was transfused with 4 units of blood because of dyspnoea at rest. Immunosuppressive treatment with high dose prednisolone 80 mg daily (1 mg/kg/day) was started and slowly reduced after 2 weeks when there was no further haemolysis and need for blood transfusion. The haemolysis recurred 4 weeks after initiation of treatment when the patient was on 40 mg daily of prednisolone. Four pulses of rituximab 375 mg/m^2^ and azathioprine 1 mg/kg/daily were then given. Despite normal thiopurine methyltransferase (TMT) enzyme levels, azathioprine caused severe neutropenia and was stopped after 2 weeks. Mycophenolate mofetil (MMF) 1 gram twice daily was started as an alternative. Over a period of 6 weeks, the haemolysis subsided and the patient became transfusion independent with normal haemoglobin levels. A CT scan done 3 months after starting MMF showed that the lymphadenopathy and splenomegaly had decreased significantly. ACE and calcium levels had normalised. The dose of steroids was tailed off 20 weeks after presentation.

## 3. Discussion

There are few reports of sarcoidosis associated with autoimmune haemolytic anaemia. In the literature, it was unclear if the association was causal or coincidental. In 1954, a case report and literature review identified 5 cases [4]. Since then, a few reports of associations between haemolytic anaemia and various types of sarcoidosis have been published [5–13]. Taken together, the case reports indicate no clear correlation between the stage of sarcoidosis and haemolytic anaemia or a poorer outcome. Haemolytic anaemia was the initial presentation in 3 of the published reports, as in our case [5–7]. Of the patients who presented with haemolytic anaemia, the underlying sarcoidosis varied from stage 0 [8] to stage 3 [6]. Others with a known diagnosis of sarcoidosis developed haemolytic anaemia later in the disease [9, 10]. In one of the case reports, the haemolysis was fatal despite treatment [11].

Evidence is poor in the literature as to the best treatments of autoimmune haemolytic anaemia and sarcoidosis. Treatment of haemolysis includes that of the cause, as in our case report. Blood transfusion is often required in severe acute cases. Transfusion may be problematic because the donor red blood cells are difficult to crossmatch and are consumed rapidly. A recent literature review recommends steroids as first line treatment for warm autoimmune haemolytic anaemia [14]. Rituximab is the preferred second line treatment; it is effective in 70–80% of cases. Another option is splenectomy in refractory cases. Other options to be considered include immunosuppressant drugs such as azathioprine and cyclophosphamide. Other treatments such as immunoglobulins and danazol have little evidence of success. High dose cyclophosphamide and alemtuzumab have been used in severe refractory cases. For cold autoimmune haemolytic anaemia, rituximab is first line.

Effective treatment of autoimmune haemolysis in sarcoid patients is dependent on a better understanding of the pathophysiological link between sarcoidosis and haemolytic anaemia. Patients with sarcoidosis have clusters of CD4+ T-helper Th1 lymphocytes in affected organs [15]. More recently, CD4+ T-helper Th17 cells were implicated in the formation of sarcoid granulomas [16]. It was hypothesised that Th1 cells are responsible for the initial autoantibody response and that the Th17 cells drive the chronic inflammation in autoimmune conditions including sarcoidosis and autoimmune haemolytic anaemia [17]. These shared immunopathogenic processes may explain why sarcoidosis and haemolytic anaemia occur together in the same patient and offer a therapeutic strategy.

Rituximab reduces the local Th17 response, which is associated with reduced inflammation and a better clinical outcome in rheumatoid patients. The inhibition of the Th17 response by rituximab was dependent on B-lymphocyte depletion [18]. A similar mechanism may apply to haemolytic anaemia and sarcoidosis. Rituximab also reduces autoantibodies to red cells by depleting the B cells. Most clinical trials have used a 4 weekly dose of 375 mg/m^2^. A meta-analysis of 21 studies found an overall response rate of 73% and a complete response rate of 42% [19]. Out of 364 patients, 22 had severe adverse events. It has been suggested that rituximab is a safer second line treatment, when compared to splenectomy.

In view that a response to rituximab often takes 8 to 16 weeks, MMF was also used in our case in order to obtain quicker immunosuppression and thus an earlier reduction of the high steroid doses. MMF has been shown to suppress both Th1 and Th17 cell subsets in several autoimmune and inflammatory disorders. Whilst MMF appears to offer no extra benefit to sarcoidosis patients compared to more established steroid-sparing agents, it was beneficial in our patient who was intolerant to azathioprine. As yet there are no studies that test the efficacy of MMF as the initial steroid-sparing agent in sarcoidosis [20].

It is not yet clear how rituximab and MMF work synergistically. The combination has been shown to be effective in a case of refractory autoimmune haemolytic anaemia but not related to sarcoidosis [21]. MMF inhibits the proliferation of T- and B-lymphocytes. The reason for both agents working so well together could be the impact on Th17 cells. As previously noted, rituximab reduces the Th17 response [18]. MMF has also been found to have a strong inhibitory effect on the Th17 cell related immune response, which could explain why the combination of rituximab and MMF is successful in treating refractory sarcoidosis and haemolytic anaemia [22].

Sarcoidosis does not always require treatment. Between 20 and 70% of patients require systemic therapy if they are at risk from or have already suffered organ damage [1]. There have been 13 trials of steroid treatment and 5 trials of immunosuppressive and cytotoxic drugs. In all these studies, there was no standard protocol for the dose and duration of steroid treatment. This may be because of the high interindividual variability in response to steroids and other immunosuppressants. Prednisolone is frequently given at a dose of 20–40 mg for 6–12 weeks. The dose is usually reduced after this. Patients often receive treatment for 12 months to prevent relapse, but it has been suggested that treatment should be stopped after 6 months. Depending on the response to treatment, it may need to be continued for several years. Due to the side effects of steroids, alternative steroid-sparing options should be considered for long-term treatment.

Although there is no established treatment for sarcoidosis complicated with haemolytic anaemia, current practice involves similar treatments for both conditions. Several of the published cases have had a good response to steroids and no further relapse at the time of being published, although their long-term follow-up was not described [5–7, 12]. In another report, the patient was treated with steroids and danazol [13]. A patient who had a splenectomy had a relapse and required long-term steroid treatment [4]. Another patient who had a splenectomy had an initial recovery but died following a haemolytic crisis one year later [11].

Sarcoidosis and autoimmune haemolytic anaemia may have a similar pathological basis involving Th1 and Th17 lymphocytes. There are no guidelines on how to treat sarcoidosis with steroid refractory haemolytic anaemia because the evidence base for this is very poor. Our patient is the first documented case of sarcoidosis complicated with haemolytic anaemia to be treated successfully with rituximab and immunosuppressive bridging with MMF. Studies based on novel immunotherapeutic strategies, including B-lymphocyte depletion, to modulate the phenotype of Th17 cells, particularly away from the Th1 phenotype, should be considered.

## Figures and Tables

**Table 1:** Blood test results on admission.

Haemoglobin	57 g/L
Reticulocytes	163 × 10^9^/L
White cell count	3.01 × 10^9^/L
Platelets	126 × 10^9^/L
LDH	1899 IU/L
Haptoglobin	<0.1 g/L
Bilirubin	101 *µ*mol/L
